# Tension band wiring versus suture anchor technique in patellar inferior pole fracture: Novel double row suture anchor technique

**DOI:** 10.1016/j.amsu.2022.104822

**Published:** 2022-11-05

**Authors:** Yong-Geun Park, Sungwook Choi, Byung Suk Kim, Seok Jae Lee, Do-Yeon Kim, Chaemoon Lim

**Affiliations:** a Department of Orthopaedic Surgery, Jeju National University Hospital, Jeju, South Korea; b Jeju National University School of Medicine, Jeju, South Korea

**Keywords:** Patella interior pole fracture, Tension band wiring, Suture anchor fixation

## Abstract

**Introduction:**

Patellar inferior pole fractures are challenging to obtain sufficient fixation. The purpose of this retrospective, case-controlled study was to compare the clinical and radiological outcomes between tension band wiring (TBW) and our novel double-row suture anchor (SA) technique in patellar inferior pole fractures.

**Materials and methods:**

This retrospective study included patients who underwent TBW or SA fixation for patellar inferior pole fractures from 2015 to 2019. A total of 63 patients were divided into two groups according to the surgical procedure: the TBW group (n = 35) and the SA fixation group (n = 28). The visual analog scale score, range of motion of the knee, Lysholm score, Kujala patellofemoral score, and patient satisfaction score were evaluated for clinical and functional outcomes. Radiological outcomes included the time to radiological union, loss of reduction, and the Insall–Salvati (IS) ratio.

**Results:**

Significant improvements in clinical outcomes were observed in both groups with no significant differences. Bone union was achieved in all patients, and there was no significant difference in the time to radiological union and the IS ratio between the two groups. All patients in the TBW group underwent additional surgeries for implant removal. However, none of the patients in the SA group underwent implant removal or experienced skin irritation.

**Conclusion:**

Our novel double-row SA technique could provide comparable fixation strength and good clinical outcomes, with fewer complications in patellar inferior pole fractures. This novel SA technique is a satisfactory alternative treatment for patellar inferior pole fractures.

## Highlights

• This novel double-row suture anchor technique provided good clinical outcomes and fixation strength comparable with tension band wiring in patellar inferior pole fractures.
• This technique has the advantage of fewer complications without the need for implant removal and is a satisfactory alternative treatment option for patellar inferior pole fractures.

## 1 Introduction

Patellar inferior pole fractures are relatively rare, accounting for 9–22.4% of all patellar fractures [1,2]. Most of these fractures are comminuted, and obtaining sufficient fixation strength is challenging [3]. The primary goal of treating patellar inferior pole fractures is to restore the knee extension mechanism. Improper treatment of these fractures may result in the loss of the knee extension mechanism and patellofemoral joint coordination [4,5]. Therefore, anatomical reduction with strong internal fixation and early rehabilitation are important for patellar inferior pole fractures. Surgical options for treating patellar inferior pole fractures include tension band wiring (TBW), wiring through cannulated screws, vertical or cerclage wiring, plate fixation, partial patellectomy, and suture anchor (SA) fixation [6]. Appropriate treatment options vary depending on the degree of comminution and the fracture pattern in patellar inferior pole fractures; previous studies on such fractures were mostly case series and lacked a comparative analysis between each surgical treatment method.

TBW is the traditional surgical method for treating patellar fractures. In particular, transverse fractures with large fragments can be easily reduced to obtain and maintain fracture fixation using TBW [7]. However, TBW is often insufficient to achieve rigid fixation due to comminuted and small fracture fragments in comminuted inferior pole fractures [8]. Despite favorable postoperative outcomes after TBW in comminuted patellar inferior pole fractures, pull-out of Kirschner wires (K-wires), reduction loss, and implant irritation have been reported as common postoperative complications [6,9]. Moreover, additional surgeries are frequently necessary for implant removal due to symptomatic implants in patellar fractures treated with TBW [6].

Recently, a novel technique using SA for patellar inferior pole fractures was introduced [10,11]. However, SA fixation often could not tolerate the pull-out strength of the knee extensor mechanism and powerful quadriceps tendon [8]. We developed a novel double-row technique using three SAs fixated on both proximal and distal fragments in patellar inferior pole fractures to overcome such pitfalls of SAs. Central threads were used for vertical tensioning, and peripheral threads were used to tighten the patellar tendon using the Krakow suture technique.

The purpose of this retrospective study was to compare the clinical and radiological outcomes between TBW and our novel double-row SA technique in patients with patellar inferior pole fractures. We hypothesized that this novel double-row SA technique could provide comparable fixation strength and satisfactory clinical outcomes in patients with patellar inferior pole fractures.

## 2 Materials and Methods

This retrospective case series study was approved by the appropriate Institutional Review Board (Ethics Committee) of under protocol number (2022-06-003). All methods were performed in accordance with the relevant guidelines and regulations (Declaration of Helsinki). The requirement for informed consent was waived by the Institutional Review Board because of the retrospective nature of the study. This study is fully complaint with the PROCESS 2020. This research is registered at Research Registry under unique identifying number: researchregistry 8263.

### 2.1 Patients

We performed a retrospective case-controlled study in general hospital from January 2015 to December 2019 in 89 patients with patellar inferior pole fractures. Between 2015 and 2017, we performed TBW for patellar inferior pole fractures. Between 2017 and 2019, SA fixation was preferred owing to its advantages reported in the literature. Postoperative clinical and radiological outcomes were evaluated at the 2-year follow-up after surgery.

**Fig. 1:**
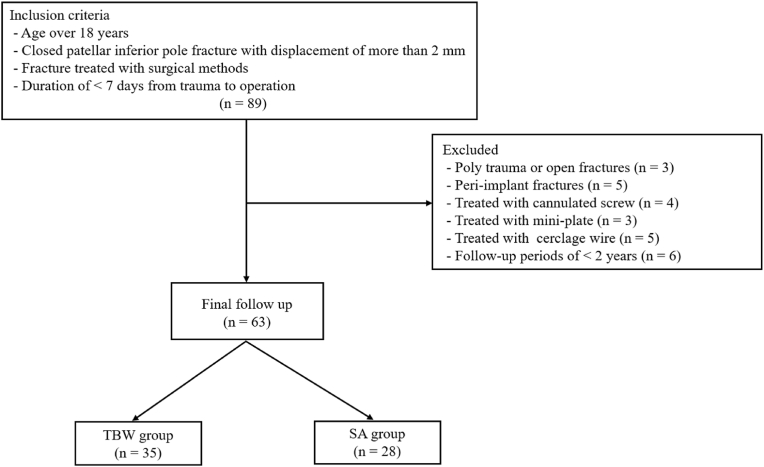
Flowchart of patient inclusion. TBW, tension band wiring; SA, suture anchor technique.

A patellar inferior pole fracture was defined as an extra-articular fracture with distal involvement of less than half of the patellar height on plain lateral knee radiographs. The inclusion criteria were as follows: (1) age >18 years; (2) closed patellar inferior pole fracture with a displacement of >2 mm; (3) fracture treated with surgical management; and (4) duration of <7 days from trauma to surgery. Patients with polytrauma or open fractures (n = 3), peri-implant fractures (n = 5), cannulated screws (n = 4), mini-plates (n = 3), cerclage wires (n = 5), or follow-up periods <2 years (n = 6) were excluded. We enrolled 63 patients with patellar inferior pole fractures treated with the TBW or the SA technique. Among them, 35 patients each were treated with TBW (TBW group), and 28 patients were treated with the SA technique (SA group) for patellar inferior pole fractures (Fig. 1). This patient size satisfied the optimal sample size calculated using the G-Power program (a free statistical program available at http://www.gpower.hhu.de/). The significance level (α), statistical power (1-β), and effect size (f) were set at 0.05, 0.8, and 0.5, respectively. There were no significant differences in baseline characteristics, such as age at operation, sex, body mass index, and the follow-up period, between the groups (Table 1).

**Table 1:** Demographics.

	TBW group (n = 35)	SA group (n = 28)	*P*
Age at operation, y	35.4 ± 15.4	34.9 ± 13.7	0.910
Gender			
Male	20 (67%)	19 (68%)	0.854
Female	10 (33%)	9 (32%)	
Side			
Right	18 (51%)	13 (46%)	0.721
Left	17 (49%)	15 (54%)	
BMI	26.8 ± 4.3	27.5 ± 2.3	0.443
Follow up period, y	2.4 ± 0.3	2.7 ± 0.7	0.059

Results expressed as mean (SD, standard deviation).

TBW, tension band wiring; SA, suture anchor technique; BMI, body mass index.

### 2.2 Surgical techniques

The surgery was performed by a single surgeon (Y.G.P) who is expert in knee trauma and sports injury. Between 2015 and 2017, we performed TBW for patellar inferior pole fractures. The SA technique was preferred between 2017 and June 2019 because of its biomechanical advantages reported in the literature. The TBW and SA techniques were similar. Surgical procedures were performed under spinal anesthesia in the supine position in both groups. A tourniquet was positioned around the proximal thigh. Prophylactic antibiotics were administered prior to tourniquet inflation. The leg was draped in a manner that allowed free movement of the knee. A radiolucent bolster was used to support the knee while maintaining a slightly flexed position. The fracture was approached through a midline longitudinal incision measuring approximately 5 cm. After the evacuation of the hematoma and interposed soft tissue, the main fracture site was identified, with preservation of any remaining retinaculum attached to the fragments.

#### 2.2.1 TBW

**Fig. 2:**
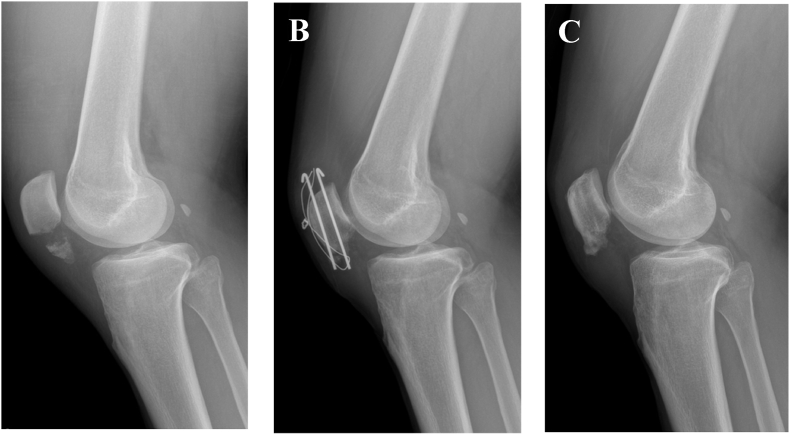
A 50-year-old man underwent tension band wiring for a left patellar inferior pole fracture. (A) Preoperative knee radiograph (B) Immediate postoperative knee radiograph (C) 24 months postoperatively.

The fractured fragments were reduced using a reduction clamp. After verifying the reduction status under fluoroscopic examination, two longitudinal K-wires were inserted parallel to each other through the reduced fracture fragments. A stainless-steel wire that could withstand the tensile force of the fracture was inserted in the figure of eight loop along the K-wires. After tightening the figure of eight wire with the knee in extension, the reduction status was confirmed under fluoroscopic examination. The proximal and distal ends of the K-wires were shortened after bilateral bending and turned towards the quadriceps tendon to prevent skin irritation and loosening. The torn medial and lateral patellar retinacula were sutured, and wound repair was performed layer-by-layer (Fig. 2).

#### 2.2.2 SA technique

**Fig. 3:**
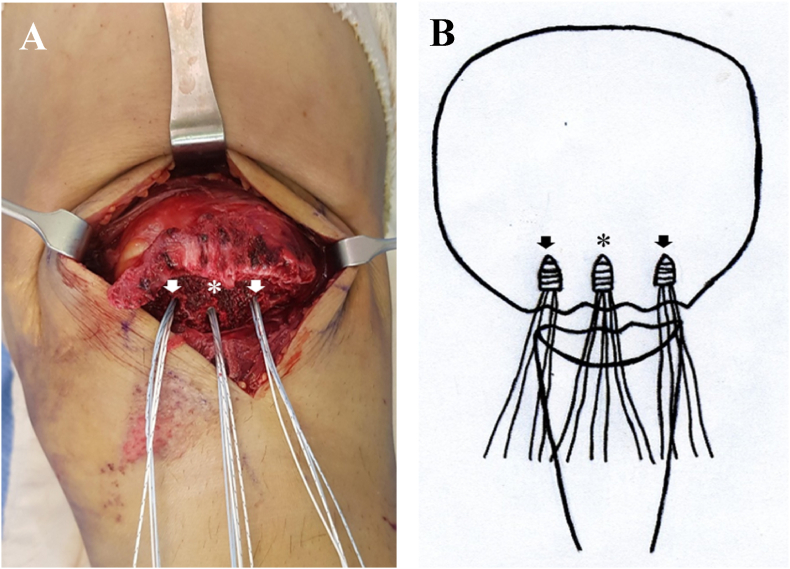
Intraoperative photograph for double row suture anchor fixation for patellar inferior pole fracture (A) The central suture anchor (asterisk) is inserted at the center of the proximal fragment. Medial and lateral suture anchors (arrow) are inserted at the medial and lateral sides of the central suture anchor. (B) Scheme of this content.

**Fig. 4:**
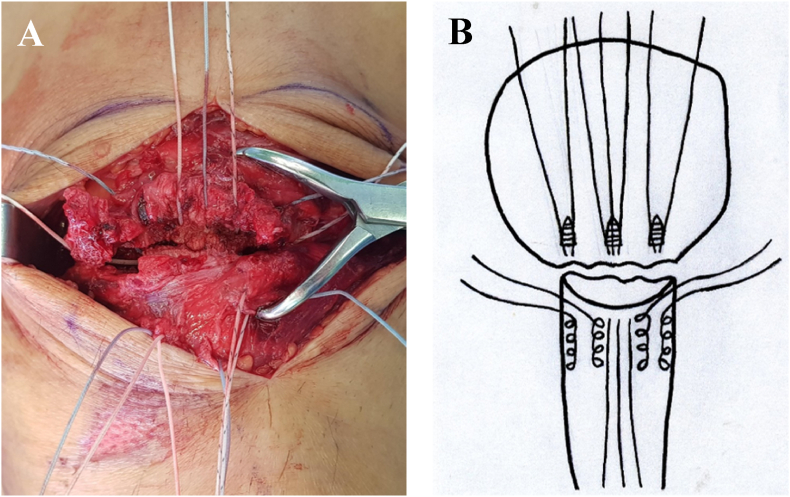
Intraoperative photograph for double row suture anchor fixation for patellar inferior pole fracture (A) The threads of the central suture anchor were tied to achieve vertical tensioning. The threads of the medial and lateral suture anchors were threaded using the Krackow suture technique through the patellar tendon. (B) Scheme of this content.

**Fig. 5:**
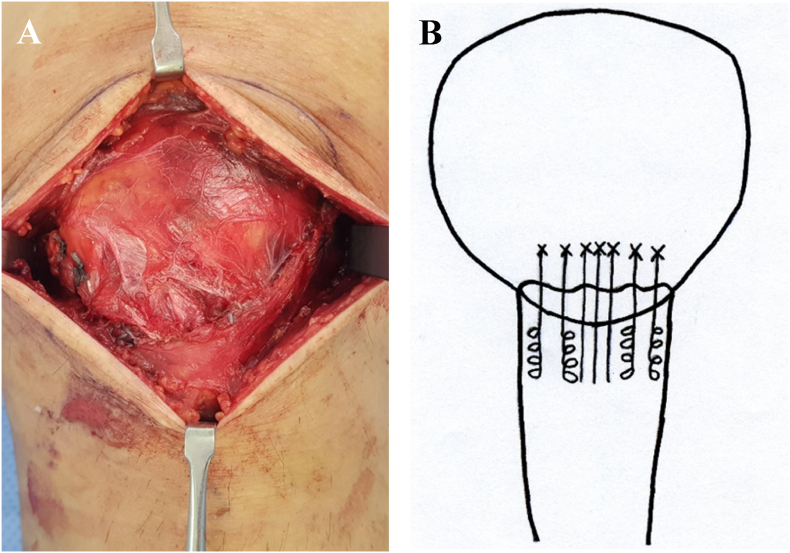
Intraoperative photograph for double row suture anchor fixation for patellar inferior pole fracture (A) The fracture is fixed with suture anchors, and the torn medial and lateral patellar retinacula are sutured. (B) Scheme of this content.

**Fig. 6:**
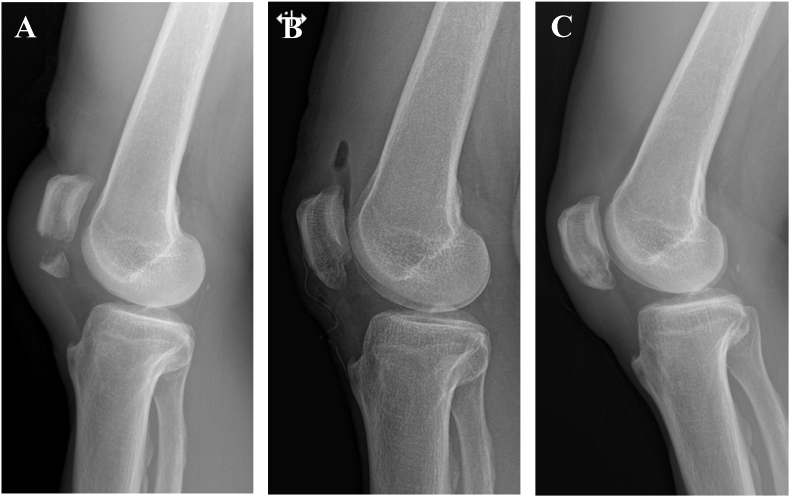
A 55-year-old man underwent suture anchor fixation for a left patellar inferior pole fracture. (A) Preoperative knee radiograph (B) Immediate postoperative knee radiograph (C) 24 months postoperatively.

A 5.5-mm absorbable SA (Twin-Fix Ti; Smith & Nephew Endoscopy, Andover, MA, USA) loaded with three ultra-braid sutures was inserted at the center of the proximal fragment of the fractured patella. Two additional 4.5-mm absorbable SAs (Twin-Fix Ti; Smith & Nephew Endoscopy, Andover, MA, USA) loaded with two ultra-braid sutures were inserted at the medial and lateral sides of the central SA (Fig. 3). After placing the SAs, the proximal and distal fragments were tunneled using the K-wire. The threads of the SAs were passed through the hole in the proximal and distal fragments (Fig. 4). The fracture fragments were reduced using a reduction clamp, and the reduction status and security of the fracture gap were verified using fluoroscopy. Upon anatomical reduction of the fracture, six threads of the central SA were tied to achieve vertical tensioning. Among the eight threads of the medial and lateral SAs, four distal threads of the distal fragment were threaded using the Krackow suture technique through the patellar tendon and tied with the proximal four threads of the proximal fragment. The torn medial and lateral patellar retinacula were sutured, and wound repair was performed layer-by-layer (Fig. 5, Fig. 6).

### 2.3 Postoperative rehabilitation

Identical postoperative management was performed for both groups. Posterior cylinder splints were applied for the first two weeks for surgical wound management, and cylinder casts were used for immobilization for two more weeks. Weight-bearing and functional quadriceps strengthening exercises, such as isometric knee extension and straight leg elevation, were encouraged from the immediate postoperative period. Progressive range of motion (ROM) of the knee joints was tolerated from the fourth postoperative week. The entire rehabilitation process was evaluated and modulated during outpatient clinic visits by the operating surgeon.

### 2.4 Clinical outcome measures

Postoperative clinical outcomes were evaluated at the 2-year follow-up. The visual analog scale (VAS) score, knee ROM, Lysholm score, Kujala patellofemoral score, and patient satisfaction score were used. Knee pain was evaluated using the VAS score. ROM of the knee was measured using a standard goniometer. The Lysholm score has been validated for patients’ specific knee symptoms, including mechanical locking, instability, pain, swelling, stair climbing, and squatting. The Kujala patellofemoral score has proven to be valid and reliable in assessing anterior knee pain and comprises 13 multiple-choice questions based on the following: limp, support, walking, stairs, squatting, running, jumping, prolonged sitting with the knee flexed, pain, swelling, patellar subluxation, atrophy of the thigh, and flexion deficiency. Patient satisfaction scores were evaluated and stratified based on a score of 1–5; very satisfied (5), somewhat satisfied (4), neither satisfied nor dissatisfied (3), somewhat dissatisfied (2), and very dissatisfied (1). Patients were initially followed up at 4 weeks, 3 months, 6 months, and 1 year and then every year thereafter.

### 2.5 Radiological outcome measures

**Fig. 7:**
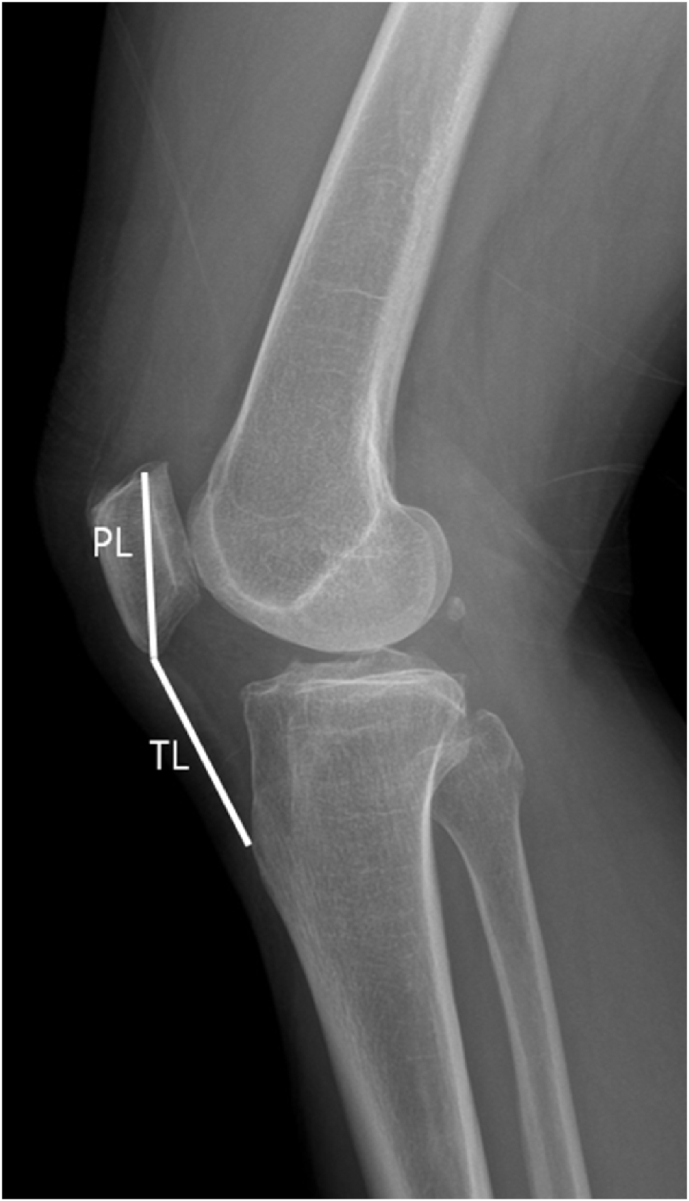
The Insall–Salvati was calculated as the greatest length of the patellar tendon (TL) divided by the length of the patella (PL).

Postoperative radiological outcomes were evaluated at the 2-year follow-up. The radiographical outcomes were measured using plain radiographs. The time to radiological union, nonunion, loss of reduction, and the Insall-Salvati (IS) ratio were measured. Radiological bone union was defined as a bridging callus crossing the fracture site on both anteroposterior and lateral plain radiographs. Nonunion was defined as a lack of fracture healing within 6 months. Loss of reduction was defined as a displacement of >3 mm compared with the immediate postoperative image. We evaluated the position of the patella using the IS ratio, which was calculated as the greatest length of the patellar tendon (TL) divided by the length of the patella (PL) (Fig. 7). In normal knees, the length of the patellar tendon is approximately equal to the length of the patella, with an average range of 0.8–1.2. Patella alta was defined as an IS ratio >1.2, and patella baja was defined as an IS ratio of <0.8 [12]. We measured the IS ratio on the immediate postoperative day and 2 years postoperatively. Potential complications, such as malunion, implant failure, soft tissue irritation, and infection, were evaluated.

### 2.6 Statistical analysis

Interobserver and intraobserver reliabilities were assessed using the intraclass correlation coefficient of the radiographical measurements, and an agreement of 0.75 was considered excellent. To determine significant differences in the VAS, ROM of the knee, Lysholm score, Kujala patellofemoral score, and radiographical parameters between the two groups, Student's *t*-test was performed. A *p*-value of ≤0.05 was considered statistically significant. Data were analyzed using the Statistical Package for the Social Sciences 19.1 (IBM, Chicago, IL, USA).

## 3 Results

### 3.1 Clinical outcomes

There was no significant difference in the clinical outcomes. The average VAS scores in the TBW and SA groups were 0.6 ± 0.9 (range, 0–3) and 0.5 ± 0.7 (range, 0–3), respectively. The average ROM in the TBW and SA groups were 128.8 ± 8.3° (range, 120–140°) and 129.6 ± 5.8° (range, 120–140°), respectively. There were no extension limitations in either of the groups. The average Lysholm scores in the TBW and SA groups were 92.4 ± 5.4 (range, 85–100) and 94.4 ± 3.4 (range, 90–100), respectively. The average Kujala patellofemoral scores in the TBW and SA groups were 87.2 ± 5.8 (range, 80–100) and 88.9 ± 3.4 (range, 82–94), respectively. There was no significant difference in the VAS (*p* = 0.058), ROM (*p* = 0.058), Lysholm score (*p* = 0.058), and Kujala patellofemoral score (*p* = 0.058) between the groups. Regarding patient satisfaction, 25 patients were very satisfied, eight patients were somewhat satisfied, and two patients were neither satisfied nor dissatisfied in the TBW group. In the SA group, 22 patients were very satisfied, five patients were somewhat satisfied, and one patient were neither satisfied nor dissatisfied. There was no significant difference in patient satisfaction (*p* = 0.058) between the groups (Table 2).

**Table 2:** Clinical outcomes.

	TBW group	SA group	*p*
VAS	0.6 ± 0.9	0.5 ± 0.7	0.446
ROM (°)	128.8 ± 8.3	129.6 ± 5.8	0.634
Lysholm score	92.4 ± 5.4	94.4 ± 3.4	0.785
Kujala patellofemoral score	87.2 ± 5.8	88.9 ± 3.4	0.856
Patient satisfaction (score)			
Very satisfied (5)	25	22	0.321
Somewhat satisfied (4)	8	5	
Neither satisfied nor dissatisfied (3)	2	1	
Somewhat dissatisfied (2)	0	0	
Very dissatisfied (1)	0	0	

Results expressed as mean ± standard deviation.

TBW, tension band wiring; SA, suture anchor technique; VAS, visual analogue scale; ROM: range of motion.

### 3.2 Radiological outcomes

Each radiographical measurement showed good to excellent interobserver and intraobserver agreements. Bone union was achieved in all patients at 14.6 ± 4.9 (range, 8–24) weeks in the TBW group and 15.2 ± 4.6 (range, 10–24) weeks in the SA group. There was no significant difference in the time to the radiological union between the two groups (*p* = 0.058).

The IS ratio of the uninjured contralateral knee was 0.95 ± 0.01 (range, 0.82–1.09) in the TBW group and 0.96 ± 0.19 (range, 0.83–1.03) in the SA group. The IS ratio of the injured knee at postoperative 2 years was 0.81 ± 0.07 (range, 0.72–0.93) in the TBW group and 0.82 ± 0.08 (range, 0.70–0.93) in the SA group. Patella baja was noted in 17 patients (48.6%) in the TBW group and 12 patients (42.9%) in the SA group (Fig. 4). There was no significant difference in the IS ratio between the two groups (*p* = 0.058). However, the IS ratio of the injured knee was significantly smaller than the IS ratio of the uninjured contralateral knee in the TBW (*p* = 0.058) and SA groups (*p* = 0.058) (Table 3).

**Table 3:** Insall-Salvati (IS) ratio.

	TBW group	SA group	*p*
Uninjured contralateral knee	0.95 ± 0.01	0.96 ± 0.19	0.658
Injured knee	0.81 ± 0.07	0.82 ± 0.08	0.852
*p*	0.021	0.05	

Results expressed as mean ± standard deviation.

TBW, tension band wiring; SA, suture anchor technique.

All patients in the TBW group underwent implant removal. Among them, 12 patients (34.3%) requested implant removal because of skin irritation. However, none of the patients in the SA group underwent implant removal or experienced skin irritation. One patient in the SA group developed a superficial wound infection that improved completely after two weeks of intravenous antibiotics.

## 4 Discussion

Obtaining firm fixation strength in patellar inferior pole fractures is challenging owing to the nature of fracture comminution and the small sizes of fracture fragments [1,2]. Among various surgical techniques, TBW and SA fixation are the commonly used methods for patellar inferior pole fractures [6]. However, due to the limited data comparing surgical outcomes between TBW and SA fixation, the current case-controlled study has its strength in evaluating the surgical outcomes of SA fixation in patellar inferior pole fractures compared with TBW. Our results demonstrated comparable clinical and radiological outcomes between SA fixation and TBW in patellar inferior pole fractures. There were no significant differences in the VAS score, ROM, Lysholm score, Kujala patellofemoral score, time to radiological union, or the IS ratio between TBW and SA fixation. Our novel double-row SA technique was able to provide sufficient fixation strength and satisfactory clinical outcomes with fewer complications without the need for additional surgery for implant removal in patellar inferior pole fractures.

TBW is the most widely used technique for the fixation of patellar fractures with large fragments. Although it is challenging to achieve rigid fixation of patellar inferior pole fractures using TBW, several studies have reported good union rates. Yang et al. reported no nonunion or loss of reduction using TBW with a cerclage wire in patellar inferior pole fractures. Despite the absence of symptomatic implants, eight patients underwent implant removal due to personal requests [13]. Chang et al. treated patellar inferior pole fractures using TBW through cannulated screws and reported no loss of reduction or implant irritation [14]. Although the rate of nonunion or loss of reduction was low using TBW in patellar inferior pole fractures, more than half of the patients underwent implant removal due to skin irritation [6]. Moreover, Smith et al. reported 22% of surgical failure rates due to soft tissue irritation caused by prominent K-wires [15]. In this study, 30 patients (85%) in the TBW group underwent implant removal, and 12 patients (34%) requested implant removal due to skin irritation. Despite satisfactory radiological outcomes using TBW, the major disadvantage associated with the technique is symptomatic implants, causing discomfort in knee ROM and skin irritation, which eventually requires additional surgeries for implant removal.

Recently, improved SA materials have provided sufficient fixation strength and have been widely used in patellar inferior pole fractures. Egol et al. reported that SA repair provided similar results to metal implants in patellar inferior pole fractures and showed fewer implant-related complications than metal implants [16]. Anand et al. used two SAs placed at the center of the proximal fragment and reported good clinical results [10]. Moreover, Kadar et al., used two SAs inserted at the center of the proximal fragment and used the Krakow suture technique for the distal fragment and patellar tendon [2]. These SA techniques achieved good fixation with fewer complications, without the need for implant removal. However, the implant failure or nonunion rate was reported to range from 7.6% to 12% in previous studies [6,16]. Despite the satisfactory reduction of fracture gaps using SA fixations, SA may be insufficient or not strong enough to withstand the tensile force on the fracture by the quadriceps tendon in previous literature.

In this study, to overcome the pitfalls of SA fixation, we introduced a novel double-row SA technique: one inserted at the center of the proximal fragment and two inserted at the medial and lateral sides of the central SA. The six threads of the central SA tied vertically play a role in load sharing, and eight threads of the medial and lateral SA grasped the patellar tendon using the modified Krackow technique. Our proposed technique has several advantages. First, in the case of comminuted patellar inferior pole fractures, rigid fixation could be achieved by vertical suturing and patellar tendon grasping using the Krackow suture technique. Second, contrary to TBW, there were no hardware-related complications such as skin irritation or discomfort, and there was no need for implant removal. Our novel double-row SA technique provided comparable fixation strength and clinical outcomes with fewer complications in patellar inferior pole fractures.

Patella baja has been reported as a potential postoperative complication of patellar fractures due to patellar tendon shortening [17]. Limitations in knee extension and anterior knee pain can be caused by patella baja [18]. Moreover, patella baja is associated with poor functional outcomes in patellar interior pole fractures based on the patellofemoral score [19]. Postoperative patella baja is reported in 12%–57% of patellar fractures [17,20]. Chang et al. reported the postoperative mean IS ratio as 0.83 ± 0.17 after SA fixation in patients with patellar inferior pole fractures [6]. Kim et al. noted that even though significant patella baja (the mean IS ratio, 0.73 ± 0.22) was caused by patellar tendon shortening after tightening the patellar tendon using a SA, there were no postoperative complications related to patella baja, such as patellofemoral pain, instability, or extension limitation of the knee joint [8]. In the current study, patella baja based on the IS ratio was noted in 17 patients (48.6%) in the TBW group and 12 patients (42.9%) in the SA group. Although the IS ratio of the injured knees was significantly reduced compared with that of the healthy contralateral knees in the SA group, there was no significant difference in the IS ratio compared with the TBW group. More importantly, there were no postoperative complications related to patella baja, which was potentially caused by patellar tendon shortening after SA fixation. Despite the insignificant clinical effect of radiological evidence of postoperative patella baja after SA fixation, it is critical to maintain the patellar tendon length during SA tightening of the patellar tendon, to avoid severe patella baja.

This study has several limitations. First, due to its retrospective nature, the study lacked randomization of each surgical treatment method into two different groups. Although there were no significant differences in the preoperative demographics between the two groups, the lack of randomization may have led to performance or selection bias. Second, the relatively small sample size limited the validity of the study for clinical practice. Third, a relatively short postoperative follow-up period may be insufficient to draw a concrete conclusion on the clinical superiority of each surgical technique in a long-term follow-up. Although a randomized controlled study comparing SA to TBW with a large sample size and long-term follow-up is needed in the future, the authors have found that SA was able to achieve firm internal fixation with favorable clinical outcomes for treating patellar inferior pole fractures. Lastly, as this study was a single center study, future large, multi-centered and prospective study are needed.

## 5 Conclusion

Our novel double-row SA technique could provide comparable fixation strength and good clinical outcomes with fewer complications in patellar inferior pole fractures. This novel SA technique is a satisfactory alternative treatment for patellar inferior pole fractures.

## Provenance and peer review

Not commissioned, externally peer-reviewed.

## Ethical approval

This retrospective study was approved by the institutional review board of our hospital (IRB No. 2022–06–003). All methods were performed in accordance with the relevant guidelines and regulations (Declaration of Helsinki).

## Source of funding

This study was supported by a research grant from the 10.13039/501100002449Jeju National University in 2020.

## Author contribution

Conceptualization, Y.G.P. and C.L.; Data curation, B.S.K., S.J.L. and D.Y.L.; Writing – original draft preparation, C.L.; Writing – review and editing, C.L. and S.C.; Supervision, S.C.; Funding acquisition, Y.G.P. All authors have read and agreed to the published version and the manuscript.

## Registration of research studies

Name of the registry: Research Registry.

Unique Identifying number or registration ID: researchregistry 8263.

Hyperlink to your specific registration (must be publicly accessible and will be checked): https://www.researchregistry.com/register-now#home/registrationdetails/63155d6463f80b002252e5f6/

## Informed consent statement

The patient's informed consent was waived by the Institutional Review Board due to the retrospective nature of the study.

## Declaration of competing interest

No potential conflict of interest relevant to this article was reported.
